# Enhancing HRQoL assessment for economic evaluation in dementia populations

**DOI:** 10.1002/trc2.70061

**Published:** 2025-03-10

**Authors:** Hannah Hussain, Anju Keetharuth, Allan Wailoo, Donna Rowen

**Affiliations:** ^1^ Sheffield Centre for Health and Related Research University of Sheffield Sheffield UK

**Keywords:** dementia, EQ‐5D, health‐related quality of life, patient‐reported outcomes, proxy report, psychometrics

## Abstract

**INTRODUCTION:**

This study aims to assess the feasibility, acceptability, and validity of EQ‐5D instrument administration methods and proxy selection for evaluating health‐related quality of life (HRQoL) in dementia populations. EQ‐5D is a widely used measure of HRQoL and is recommended by the National Institute for Health and Care Excellence for cost‐effectiveness analyses of health interventions.

**METHODS:**

Individual‐level data from three trials were analyzed separately to evaluate missing data rates, inter‐rater agreement, responsiveness, and predictors of EQ‐5D (EQ‐5D‐3L and EQ‐5D‐5L) dimensions and index values. The study used psychometric analyses, correlations, and multivariate linear regression models to evaluate EQ‐5D dimension reports. Reports from both people with dementia (PwD) and proxies were compared to assess reliability across different settings and proxy types.

**RESULTS:**

Proxy‐reported EQ‐5D achieved higher completion rates compared to reports from PwD, with proxies showing greater responsiveness to changes in symptom scores over time. Face‐to‐face instrument administration for informal proxies was favored over postal methods, and proxy selection was found to be crucial, with informal proxies recommended for community‐dwelling PwD and staff proxies for institutionalized populations. Inter‐rater agreement was strongest for the “mobility” dimension, with differences in reporting by dimension. Novel guidelines on integrating EQ‐5D data reported by PwD and proxies are proposed. Combining self‐ and proxy‐reported data to generate an integrated utility score potentially reflects a more holistic perspective and may enhance the accuracy of HRQoL assessment, compared to relying solely on one respondent's reports.

**DISCUSSION:**

The importance of careful administration and proxy selection for HRQoL data collection and application in dementia trials and studies is highlighted. These findings have implications for informing economic evaluations of dementia interventions, emphasizing the potential need for tailoring approaches to HRQoL assessment based on the residential status of the PwD.

**Highlights:**

- The EQ‐5D is a widely used measure in dementia trials, but challenges like missing data and discrepancies in inter‐rater agreement highlight complexities in assessing health‐related quality of life (HRQoL), especially in advanced stages.
- This study empirically examines the feasibility, acceptability, and validity of EQ‐5D data from people with dementia and proxies, using individual‐level data from three trials.
- Recommendations are provided to improve data collection practices, enhancing the reliability of HRQoL assessments in clinical trials for dementia interventions.
- Optimized proxy selection criteria and administration methods tailored to residential status can improve HRQoL assessments, supporting more accurate economic evaluations and better‐informed care decisions.

## 1 BACKGROUND

Dementia is a complex neurodegenerative condition, requiring validated instruments to assess core outcomes including cognition, behavior/mood, and function.^1^, ^2^, ^3^

Health‐related quality of life (HRQoL) instruments play a pivotal role in dementia research. Researchers must choose between dementia‐specific measures, like the Dementia Quality of Life (DEMQOL) scale^4^ and the Quality of Life in Alzheimer's Disease (QOL‐AD) scale,^5^ which capture dementia‐specific experiences, and generic instruments, like EQ‐5D, which offer broader comparability but may lack detail. There are additional measures such as DEMQOL Utility Score (DEMQOL‐U) and Alzheimer's Disease Five Dimensions (AD‐5D), but EQ‐5D remains the focus of this study due to its widespread use in economic evaluations.

EQ‐5D is recommended by the National Institute for Health and Care Excellence (NICE)^6^ for use in economic evaluations in the UK. Despite its broad use, EQ‐5D faces challenges in reliability and consistency with dementia populations. While EQ‐5D is feasible and acceptable in this context, these limitations highlight the importance of further investigation to ensure its suitability and validity for HRQoL assessment in dementia populations.^7^, ^8^, ^9^ Therefore, EQ‐5D (EQ‐5D‐3L and EQ‐5D‐5L) remains the focus of this study. Our previous systematic review examined the convergent validity of EQ‐5D, focusing on relationships between EQ‐5D scores and dementia symptom outcomes.^1^ While some correlations were identified, evidence specific to individual EQ‐5D dimensions is limited.

NICE's reference case states that HRQoL should be provided by patients, but proxy reports are acceptable when direct collection is not possible.^10^ Challenges in collecting HRQoL data from people with dementia (PwD) stem from cognitive impairments, leading to reliance on proxy assessments.^9^ Proxies can be informal (family or friends) or formal (caregivers or professionals). Differences between self and proxy assessments are recognized, with PwD often providing more optimistic HRQoL reports.^1^, ^9^, ^11^ Recognizing that comprehensive HRQoL assessment includes both objective and subjective aspects of health,^12^ high‐quality, accurate HRQoL data are essential for analyses. Additional challenges include instrument administration methods and proxy selection, based on PwD cognitive impairment and residential status. Currently, no guidelines exist for HRQoL data collection in dementia populations.

The primary aim of this study is to provide targeted recommendations for optimizing the collection of HRQoL data in populations with dementia. To achieve this, the study examines the feasibility, acceptability, and suitability of EQ‐5D administration and proxy reporting in dementia contexts by analyzing individual‐level data from existing dementia trials that collected EQ‐5D. This analysis seeks to explore the specific challenges associated with HRQoL data collection in dementia populations via the EQ‐5D instrument, addressing gaps identified in our previous systematic review, which found limited evidence at the EQ‐5D dimension level. The study is guided by the following research questions:

1. Feasibility and acceptability issues:
  Analysis: Evaluate missing data, administration methods, and floor/ceiling effects to assess data collection processes.
2. Discrepancies between PwD and proxy reports:
  Analysis: Inter‐rater agreement analysis to identify discrepancies between PwD and proxy reports across EQ‐5D dimensions.
3. Factors influencing EQ‐5D utility scores:
  Analysis: Multivariate linear regression to determine factors influencing EQ‐5D utility scores and variations in HRQoL data.
4. Responsiveness to changes in dementia symptoms:
  Analysis: Evaluate sensitivity of PwD and proxy reports to symptom changes over time.

Together, these analyses aim to address the overarching goal of optimizing HRQoL data collection methods for dementia populations, contributing to a more nuanced understanding of HRQoL assessment in dementia research.

## 2 METHODS

### 2.1 Datasets

This study used data from three dementia studies: the Reminiscence Groups for People with Dementia and their Family Caregivers (REMCARE) trial,^13^ the Access to Timely Formal Dementia Care in Europe (ACTIFCARE) cohort study,^14^ and the Enhancing Person Centred Care in Care Homes (EPIC) trial;^15^ details are available in the referenced articles. Table 1 summarizes the key characteristics.

**RESEARCH IN CONTEXT**

1. **Systematic review**: We conducted two previous systematic reviews of existing literature on the topic, focusing on studies evaluating EQ‐5D for health‐related quality of life (HRQoL) assessment in dementia populations. While EQ‐5D is established as suitable for dementia populations, challenges in self and proxy reporting persist, producing differences in reports and uncertainty on which respondent's reports to rely on for use in evaluations.
2. **Interpretation**: Our research advances the field by empirically examining EQ‐5D data from three trials, offering actionable insights into improving HRQoL data collection by recommending optimal administration methods and proxy selection criteria.
3. **Future directions**: Future research should explore the application of a proposed hybrid utility score, potentially representing a more accurate HRQoL assessment. Further studies to address challenges in accurately assessing subjective dimensions like “pain/discomfort” and “anxiety/depression,” particularly for severe dementia stages, are warranted. Additionally, qualitative research could offer insights into the optimal respondent selection and accuracy of reporting.

**TABLE 1:** Summary of key trial characteristics for three dementia studies.

Study name	REMCARE	ACTIFCARE	EPIC
Study type	RCT of non‐pharmacological intervention	Prospective cohort study for best‐practice development	RCT of non‐pharmacological intervention
PwD recruited (*n*)	488	451	734
Residential status of PwD	Community dwelling		Institutionalized
Version of EQ‐5D	EQ‐5D‐3L	EQ‐5D‐5L	
Participants recruited	PwD and informal proxy		PwD, informal, and staff proxy
Dementia severity stage^a^	Mild to moderate		Mild to severe
Longitudinal EQ‐5D data	T0 (baseline), T1 (3 months), T2 (10 months)	T0 (baseline), T1 (6 months), T2 (12 months)	T0 (baseline), T1 (6 months), T2 (16 months)
Measures core dementia symptom measures		Included cognition	
	Included a measure of function		
	Included a measure of behavior/mood		

Abbreviations: ACTIFCARE, Access to Timely Formal Dementia Care in Europe; CDR, Clinical Dementia Rating; EPIC, Enhancing Person Centred Care in Care Homes; PwD, person with dementia; REMCARE, Reminiscence Groups for People with Dementia and their Family Caregivers; QoL, quality of life; RCT, randomized controlled trial; T0, baseline; T1, first follow‐up; T2, final follow‐up.

^a^ Dementia severity stage of sample defined by CDR at T0.

EQ‐5D, a commonly used generic measure of HRQoL, has two versions: EQ‐5D‐3L and EQ‐5D‐5L. They differ in the number of response options for each item, with three and five levels, respectively (a full description of EQ‐5D can be found in the supporting information). All analyses were stratified by EQ‐5D version (3 and 5L). Analyses were not combined due to distinct response structures. ACTIFCARE and EPIC collected EQ‐5D‐5L data; REMCARE collected EQ‐5D‐3L. Demographic data and Clinical Dementia Rating (CDR) scale stage were recorded, with follow‐up CDR data available for ACTIFCARE and EPIC. The CDR is measured by clinicians to determine the person's dementia stage (whereby stages are: [CDR 0/0.5] questionable, [CDR 1] mild, [CDR 2] moderate, and severe [CDR 3]^16^]. EPIC assessed PwD living in care settings, including care homes and other facilities, hereafter referred to as institutionalized populations; REMCARE and ACTIFCARE assessed community‐dwelling PwD. Detailed summary statistics can be found in Table S1 in supporting information.

### 2.2 Proxy assessment details

Table S1 presents informal proxy relationships to PwD for each study. In the EPIC trial, proxy‐reported EQ‐5D was collected using face‐to‐face or telephone interviews, or postal self‐report. Tables S2 and S3 in supporting information show counts for different administration methods and counts by proxy relationship. The ACTIFCARE study collected informal proxy visit frequency (see Table S4 supporting information).

### 2.3 Statistical analyses

#### 2.3.1 Feasibility and acceptability

Feasibility and acceptability were examined by missing data percentages. While missing data can provide insights into these properties, it is not a comprehensive measure. Other factors related to completion challenges in this population, such as cognitive load or task fatigue, would offer a more complete picture. However, as this was a secondary data analysis, we had limited access to such variables. We acknowledge this limitation while leveraging the available data to generate meaningful insights. Independent samples *t* tests assessed differences in dementia severity (via CDR) between PwD with and without missing data. EQ‐5D dimension‐level floor and ceiling effects are also analyzed, using common dementia study thresholds: > 60% for “no problems” and > 15% for “extreme problems.”^17^

### 2.4 Inter‐rater agreement

Inter‐rater agreement between PwD and proxy EQ‐5D reports was assessed using a baseline cross‐section of EQ‐5D‐5L data. Each EQ‐5D dimension was analyzed separately to ensure that the unique properties of each dimension were appropriately examined. Analyses were performed at the dimension level using linear weighted Kappa coefficients and the percentage of exact agreement. CDR stages were used to stratify reports to explore how agreement varied with dementia severity. Thresholds for interpretation of agreement were applied: < 0 indicates poor, 0 to 0.2 indicates slight, 0.21 to 0.4 indicates fair, 0.41 to 0.6 indicates moderate, and > 0.6 indicates strong agreement.^18^ These thresholds reflect agreement beyond chance, as measured by the Kappa coefficient, and are distinct from the percentage of exact agreement, which represents the proportion of identical responses between PwD and proxies. Intra‐class correlation coefficients (ICCs) using one‐way random‐effects analysis of variance (ANOVA) compared agreement between EQ‐5D index scores. A minimum ICC of 0.7 is recommended for group comparisons of inter‐rater agreement.^19^ Additionally, Spearman correlation was used to assess the relationship between ordinal data, particularly when examining trends in agreement across EQ‐5D dimensions and respondent types.

### 2.5 Factors influencing EQ‐5D index scores

Multivariate linear regression analyses explored the impact of demographic and clinical variables on EQ‐5D reports. The analysis had two objectives: to explore factors influencing EQ‐5D index scores, including demographic and clinical predictors, and to evaluate the suitability of different proxy types for reporting on behalf of PwD. Selected variables, including demographic factors such as sex and clinical scores such as dementia severity (CDR stages), were included based on their established importance in influencing HRQoL and proxy reporting tendences in dementia populations. These variables reflect a balance between the scope of the analysis and the limitations of secondary data access. This exploratory analysis aimed to identify the most suitable (informal) proxy type, to ensure accuracy and reliability. While multivariate linear regression may not align with normality assumptions of EQ‐5D's distribution, its application serves to identify important variables and their directional impact, rather than for predictive purposes. Acknowledging this limitation, the choice of linear regression is deemed suitable for the exploratory nature of this analysis. Results of this analysis are provided in the supporting information.

### 2.6 Responsiveness

Psychometric analyses evaluated EQ‐5D dimension report validity, aligning with symptom changes over time. Baseline (T0) and final follow‐up (T2) data from EQ‐5D‐5L datasets were used. The direction of reporting was examined alongside changes in dementia symptom measure scores over time by assessing counts and proportions of PwD–proxy dyads reporting dimension‐level changes in the following directions: no change, improvement, and worsening. The dementia symptom measures used for comparison to EQ‐5D dimensions are identified and referenced in a previous systematic review.^1^ A summary of this evidence can be found in Table S5 supporting information. Effect size calculations were performed using the Cohen D equation:

$$\mathrm{Cohe}n\;D=\frac{T2\;\mathrm{mean}-\;T0\;\mathrm{mean}}{\mathrm{pooled}\;\mathrm{standard}\;\mathrm{deviation}\;\mathrm{of}\;\mathrm{the}\;\mathrm{change}}$$

Effect sizes reflect change direction and magnitude, with thresholds of 0.2 for small effect, 0.5 for moderate effect, and 0.8 for large effects.^20^ Changes in EQ‐5D assessments were correlated with the change in dementia symptom measure scores using Pearson correlation. This method was chosen to assess the strength of linear relationships between variables. While Pearson correlation assumes normally distributed data and linearity, we acknowledge that EQ‐5D index scores may deviate from these assumptions. Results were interpreted with caution within the context of exploratory analysis. Responsiveness analyses of utility scores considered mean scores from all studies. EQ‐5D‐3L and EQ‐5D‐5L dimension score reports were never merged due to reporting level differences. Analyses were conducted separately for each dataset due to differing dementia symptom measures, preventing outcome score merging.

## 3 RESULTS

### 3.1 Feasibility and acceptability

Table 2 presents missing data, EQ‐5D index scores, and floor/ceiling effects. Proxies across all studies had higher proportions of complete EQ‐5D reports than PwD. Proxy‐reported EQ‐5D had less missing item data, especially staff‐proxy reports with no dimensions missing more than two missing observations. The “usual activities” dimension had the most missing data across studies and respondents, followed by “anxiety/depression,” highlighting potential challenges for respondents when reporting these specific dimensions.

**TABLE 2:** Feasibility and acceptability of EQ‐5D instrument.

Study	REMCARE (EQ‐5D‐3L)		ACTIFCARE (EQ‐5D‐5L)		EPIC (EQ‐5D‐5L)		
Respondent	PwD	Proxy	PwD	Proxy	PwD	Proxy	Staff
Complete EQ‐5D instruments, *n* (%)	1153 (80.7)	1206 (83.0)	1105 (83.8)	1182 (88.2)	765 (34.6)	349 (77.0)	1696 (77.2)
Index score, mean (SD)	0.75 (0.25)	0.59 (0.28)	0.77 (0.19)	0.62 (0.23)	0.82 (0.24)	0.33 (0.38)	0.52 (0.39)
Proportion missing, floor and ceiling effect, %							
**Mobility**							
missing	0.3	0	<0.1	0	2.6	1.1	<0.1
*Floor effect*	0.3	0.3	0.7	2.0	9.0	30.5	33.3
*Ceiling effect*	65.5	49.7	56.3	39.3	57.6	15.4	34.7
**Self‐care**							
missing	0.4	0	0.8	<0.1	2.9	1.7	<0.1
*Floor effect*	2.1	6.2	2.0	5.5	2.4	57.7	53.2
*Ceiling effect*	82.1	50.0	75.4	43.8	67.2	6.1	13.4
**Usual activities**							
missing	2.0	0.4	2.2	0.3	4.8	1.7	0
*Floor effect*	4.1	21.0	1.9	9.4	2.4	48.5	15.4
*Ceiling effect*	68.9	22.7	57.9	20.8	76.5	19.7	62.6
**Pain/discomfort**							
missing	0.5	0.2	0.8	0.3	2.2	1.4	<0.1
*Floor effect*	4.2	6.6	0.6	0.9	0.4	1.7	0.2
*Ceiling effect*	55.8	41.3	57.2	36.9	71.9	43.9	74.3
**Anxiety/depression**							
missing	1.3	0.3	0.9	0.7	4.0	0.9	<0.1
*Floor effect*	2.0	5.5	0.3	0.4	0.6	2.2	0.3
*Ceiling effect*	63.3	38.7	59.5	38.5	77.5	50.0	75.7

Missing data by dementia severity stage, %						
	Missing all EQ‐5D items			Missing at least one EQ‐5D item		
	PwD	Informal proxy	Staff proxy	PwD	Informal proxy	Staff proxy
**CDR stage**						
Very mild (0.5)	<1	16.7	<1	1.6	8.7	1.7
Mild (1)	11.1	83.3	10.1	14.2	39.1	11.1
Moderate (2)	37.3	–	37.0	37.8	30.4	37.3
Severe (3)	51.0	–	52.1	46.4	21.8	49.9

Missing data by informal proxy relationship to PwD, %^a^		
	Complete EQ‐5D reports, *n* (%)	Missing at least one item, *n* (%)
**Relationship**		
Spouse/partner	1699 (62.2)	25 (48.0)
Adult child	837 (30.6)	14 (27.0)
Other family member	97 (3.6)	7 (13.4)
Friend/neighbor	99 (3.6)	6 (11.6)
Total	2732	52

Missing data by administration method, *n* %^a^		
	Complete EQ‐5D reports, *n* (%)	Missing at least one EQ‐5D item, *n* (%)
**Administration method**		
Face‐to‐face	122 (35.3)	1 (4.2)
Telephone	21 (6.0)	–
Postal	203 (58.7)	23 (95.8)
Total	346^*^	24

Abbreviations: ACTIFCARE, Access to Timely Formal Dementia Care in Europe; CDR, Clinical Dementia Rating; EPIC, Enhancing Person Centred Care in Care Homes; Proxy, informal proxy; PwD, person with dementia; REMCARE, Reminiscence Groups for People with Dementia and their Family Caregivers; SD, standard deviation; Staff, staff proxy.

^a^ Missing at least one EQ‐5D dimension report, not including cases with complete missing data.

Missing data increased with dementia severity, with 51% missing EQ‐5D items in severe cases. An independent samples *t* test showed significantly worse dementia severity in PwD with missing EQ‐5D compared to those with complete data (2.42 vs. 1.44, *P* < 0.0001). Most missing dimension‐level data from proxies were attributed to spouse/partner and offspring proxy reports, the predominant type of informal proxy types recruited (see Table S1). Postal administration accounted for many instances in which proxies omitted item responses.

Ceiling effects were observed across all EQ‐5D dimension reports by PwD. Notable discrepancies between PwD and proxy assessments were observed in the EPIC trial data, especially for “self‐care” and “usual activities” dimensions with informal proxies. PwD self‐reports showed more “no problems” compared to proxy reports, which indicated severe impairments. Similarly, for “usual activities,” ~ 77% of PwD reports indicated “no problems,” compared to nearly 50% of informal proxies reporting the worst impairment level. Informal proxy reports aligned more closely with staff proxy reports for “self‐care,” while staff proxy assessments more closely resembled PwD reports for “usual activities.”

### 3.2 Inter‐rater agreement

Table S6 in supporting information presents correlations between PwD and proxy EQ‐5D‐5L dimension reports by CDR stage, showing decreasing association strength with increasing dementia severity.

Comparative analysis of PwD and proxy reports on EQ‐5D dimensions show that “mobility” had the strongest agreement, particularly in dyads with mild dementia. “Self‐care” demonstrated moderate agreement between PwD–informal proxy dyads, decreasing with increased dementia severity. PwD–staff proxy dyads had lower agreement rates for “self‐care” (~ 20%), with negligible Kappa coefficients (*k* < 0.1). “Usual activities” yielded the lowest exact agreement for PwD–informal proxy dyads (36%), with a Kappa coefficient of 0.26. PwD–staff proxy dyads showed higher exact agreement (59%) for “usual activities” reports, but poor agreement as indicated by a Kappa coefficient of 0.15.

The ICC for inter‐rater agreement of index scores showed poor agreement (*r* = 0.35) for PwD–informal proxy dyads. The ICC was slightly stronger in mild dementia dyads and had a narrower confidence interval than moderate and severe stages. For PwD–staff proxy dyads, the ICC also reflected poor agreement, strongest moderate dementia dyads, though the confidence interval included zero, indicating that correlation in moderate dementia may not be statistically significantly different from other stages. Inter‐rater agreement results are presented in Table 3.

**TABLE 3:** Inter‐rater agreement between PwD–proxy dyads.

EQ‐5D‐5L	% of exact agreement	Kappa coefficient			
		Sum	Mild	Moderate	Severe
*PwD–informal proxy dyad*					
Mobility	49.6	0.55	0.60	0.53	0.04
Self‐care	51.1	0.42	0.45	0.28	0.04
Usual activities	36.2	0.26	0.28	0.20	0.05
Pain/discomfort	51.0	0.42	0.43	0.35	0.61
Anxiety/depression	43.1	0.30	0.30	0.36	−0.13
Index score^a^	−	0.35 *[0.31–0.38]*	0.36 *[0.31–0.41]*	0.18 *[0.08–0.27]*	0.10 *[0.00–0.44]*
*PwD–staff proxy dyad*					
Mobility	43.0	0.31	0.52	0.26	0.08
Self‐care	20.1	0.05	0.09	0.03	0.01
Usual activities	59.4	0.15	0.23	0.17	0.11
Pain/discomfort	57.4	0.16	0.21	0.20	−0.08
Anxiety/depression	56.5	0.20	0.30	0.15	0.14
Index score^a^	−	0.14 *[0.01–0.25]*	0.05 *[0.00–0.22]*	0.21 *[0.00 – 0.36]*	0.00 *[0.00–0.05]*

Abbreviations: CDR, Clinical Dementia Rating; ICC, intra‐class correlation; PwD, person with dementia.

^a^ ICC for EQ‐5D index scores; dementia severity defined by CDR stage.

### 3.3 Responsiveness

Table S7 in supporting information presents the relationship between EQ‐5D‐5L dimension reports and changes in dementia symptom scores over time. Proxy reports more effectively captured deteriorating functional status than self‐reports. Staff proxies were most likely to report no change in EQ‐5D‐5L dimensions over time when the PwD's functional status remained stable. Similar patterns emerged for behavior/mood status, with staff proxies demonstrating the most consistent reporting with symptom score changes.

PwD‐reported improvements in “usual activities” often coincided with worsening functional status. For “pain/discomfort,” both PwD and informal proxy reports of worsening were frequently associated with declining functional abilities, contrasting with staff proxies. Staff proxies were more likely to report improvements in “pain/discomfort” when corresponding behavior or mood improvements were observed. For “anxiety/depression,” staff proxy reports of worsening or improvement often correlated with corresponding behavior/mood reports. Associations with cognition were only available from the REMCARE study, indicating greater alignment between worsened “anxiety/depression” and deteriorated cognition, particularly in PwD reports compared to informal proxies.

Responsiveness results via effect size estimates are presented in Table 4 for EQ‐5D dimensions. Staff proxy reports often yielded larger effect sizes than those from PwD or informal proxies, particularly for “mobility” and “self‐care” dimensions. Correlations between EQ‐5D report changes and symptom score changes are reported in Table S8a supporting information, revealing larger coefficients for proxy reports, especially regarding functional measure changes.

**TABLE 4:** Results of responsiveness analysis.

Effect size estimates	CDR		Function			Neuropsychiatric symptoms			Depression^a^		MMSE	
Change in EQ‐5D dimension reports by different respondents	Stable	Worse	Stable	Worse	Better	Stable	Worse	Better	Worse	Better	Worse	Better
*Mobility*												
PwD	−0.15	−0.34	*−0.36*	*−0.11*	*0.09*	*−0.23*	−0.07	−0.44	0.02	−0.02		
Informal proxy	−0.11	−0.36	−0.13	−0.21	0.14	−0.04	*−0.24*	−0.17	−0.14	−0.02		
Staff proxy	−0.29	−0.56^a^	−–0.49	−0.49	−	−0.09	−0.41	*−0.33*	−	−		
*Self‐care*												
PwD	*−0.17*	−0.36	*−0.22*	*−0.19*	*0.04*	−0.11	−0.20	−0.22				
Informal proxy	−0.24	−0.66^*^	−0.05	−0.49	0.02	−0.12	−0.43	−0.31				
Staff proxy	−0.17	*−0.72* ^a^	−0.22	−0.61^a^	−	−0.11	*−0.55*	−0.24				
*Usual activities*												
PwD	*0*	−0.11	*−0.08*	*0*	*0.04*	0.01	−0.01	−0.01				
Informal proxy	−0.22	−0.66^a^	−0.10	−0.33	−0.04	−0.39	−0.33	−0.22				
Staff proxy	−0.11	*−0.64* ^a^	−0.22	−0.33	−	0.01	−0.44	−0.17				
*Pain/discomfort*												
PwD	*0.04*	−0.03	*0.06*	*0.06*	*0.05*	0.44	−0.01	−0.00	0.17	0.14		
Informal proxy	−0.03	−0.07	−0.04	−0.03	0.06	0.01	−0.08	0.01	−0.11	−0.04		
Staff proxy	−0.04	*0.22*	0.04	0.11	−	0.44	−0.15	0.22	−	−		
Anxiety/depression												
PwD	0	0.16				0.12	0	0.05	0.11	0.07	−0.01	0.06
Informal proxy	0.06	0.16				−0.16	−0.19	0.13	−0.19	0.24	0.05	−0.07
Staff proxy	0.13	0.22				0.33	0.33	0.44			−	−

Abbreviations: CDR, Clinical Dementia Rating; MMSE, Mini‐Mental State Examination; PwD, people with dementia.

^a^ Using EQ‐5D‐3L data from Reminiscence Groups for People with Dementia and their Family Caregivers (REMCARE) trial dataset.

“Usual activities” and “anxiety/depression” dimensions showed more effective change alignment with symptom scores when measured by proxy reports. However, responsiveness related to “pain/discomfort” elicited low effect sizes across respondent types, with negligible correlation coefficients. For “anxiety/depression,” correlation with cognition exhibited a negative trend for PwD reports, suggesting improved cognitive states may influence more favorable “anxiety/depression” responses.

### 3.4 Factors influencing EQ‐5D index scores

Regression analyses indicate that proxy models explain more variance in EQ‐5D scores than PwD models. Key predictors include functional measures and proxy demographics, such as sex. Detailed results and interpretation are available in Tables S9a and S9b in supporting information.

## 4 DISCUSSION

Our study highlights the importance of administration methods and proxy selection in HRQoL data collection in dementia populations via analysis of EQ‐5D administration. Significant discrepancies were found between PwD and proxy reports, particularly in more observable EQ‐5D dimensions. This aligns with recent research by Buchholz et al., analyzing discrepancies between self and proxy EQ‐5D reports in dementia populations using a different trial‐based dataset.^21^ Both studies identified “usual activities” as having the lowest inter‐rater agreement, reinforcing this aspect of EQ‐5D as challenging to interpret. Relying solely on proxy repots may not be appropriate for all EQ‐5D dimensions, suggesting a combination of self‐ and proxy‐reported dimensions may provide a more accurate assessment. This approach uses strengths of both respondent types and mitigates discrepancies observed in certain dimensions. Findings on proxy selection and administration methods may also apply to other HRQoL instruments, such as DEMQOL‐U and AD‐5D. However, the unique properties of these instruments may lead to different implications, warranting further investigation. It is important to note that the recommendations made in this study are tailored specifically to EQ‐5D. While certain findings may have broader relevance, their applicability should be assessed in the context of each instrument's unique characteristics and design.

### 4.1 Administration method

Administration method is crucial when obtaining proxy reports. Most missing EQ‐5D data were obtained via postal administration. It is recommended that EQ‐5D be administered in person to ensure completion of the same day, which is critical when comparing PwD and proxy assessments. This is important in dementia, as symptom progression is unpredictable and cognitive decline is complex—not necessarily occurring in a linear fashion.^22^ In‐person completion allows for clarifications if required, potentially minimizing missing data due to item interpretation difficulties. Focusing on data quality is key to ensure its usability in later analyses.

### 4.2 Proxy selection

Obtaining three sets of EQ‐5D reports (self‐report, informal proxy, staff proxy) capturing a single individual's health status poses significant challenges, as evidenced by the low enrolment and high attrition rates of informal proxies in the EPIC trial. Focusing on selecting the most appropriate proxy and obtaining high‐quality data from both the proxy and PwD is more advantageous. It is recommended to use informal proxies for community‐dwelling PwD, while staff proxies are recommended for institutionalized dementia populations. Staff proxy data exhibited lower missing data rates and closely aligned with dementia symptom score changes. Staff proxy reports demonstrated greater sensitivity to stable symptom scores, likely due to their clinical training and prior knowledge.

The primary determinant for proxy selection should be close and current awareness of the PwD's condition, influenced by contact frequency.^23^ Staff proxies typically have daily contact with the PwD and are more suitable for institutionalized populations than visiting proxies. For community‐dwelling PwD, spouses/partners may be preferable proxies over other family members, like offspring. Spouse/partner proxies exhibited larger coefficients in EQ‐5D index score predictors, indicating a stronger association with clinical factors of CDR stage and Mini‐Mental State Examination score. Spouses/partners are more likely to live with the PwD, resulting in closer and more frequent contact, enhancing daily awareness of the PwD's health status.

Inter‐rater agreement was strongest for the “mobility” dimension, evidenced by both correlation and Kappa coefficients. This dimension also had the highest exact agreement percentage, particularly with informal proxies. Conversely, inter‐rater agreement was low for “self‐care” between PwD and staff proxies, with minimal Kappa and Spearman rank coefficients. This disparity highlights inter‐proxy variability and the potential impact of proxy characteristics on HRQoL assessments. Poor agreement among staff proxies for the “self‐care” dimension may be attributed to caregiving burden,^24^ and the ceiling effects observed with PwD reports of this dimension (≈ 67% of PwD reported no problems with “self‐care” in EPIC). It is reasonable to expect PwD in this trial's population to experience self‐care issues; therefore, differences between PwD and proxy reports do not necessarily imply deficiencies in proxy responses.

### 4.3 Indication of a proxy assessor

Correlations between EQ‐5D dimension reports revealed more robust inter‐rater agreement in mild dementia stages, with disagreement increasing as dementia severity progressed; coupled with the finding that missing EQ‐5D data tended to be in more severe dementia stages, suggests that PwD are capable of reliable self‐reporting in the milder stages. Prior literature has also highlighted the feasibility and acceptability of EQ‐5D assessment in early stages,^17^, ^25^, ^26^ potentially retracting the need for proxy reliance. However, as dementia progresses, proxy input becomes increasingly vital.

### 4.4 Combining EQ‐5D dimension reports by respondent type

The responsiveness of EQ‐5D‐5L reports was assessed to determine how well respondent dimension scores aligned with changes in dementia symptom measures. For “mobility,” informal proxy reports often contradicted functional outcomes, indicating validity issues, whereas staff proxy reports demonstrated moderate responsiveness and outperformed both informal proxy and PwD reports. For “self‐care,” proxy reports exhibited stronger responsiveness than PwD reports, with staff proxies showing the most substantial effect sizes. Both informal and staff proxy reports for “usual activities” aligned more closely with symptom changes compared to PwD reports.

Findings were inconclusive across respondent types for “pain/discomfort,” with mixed patterns in reporting health status changes. Weak responsiveness was observed across all respondent types, indicating challenges in capturing this dimension accurately. Responsiveness for “anxiety/depression” was minimal across respondents. Although “anxiety/depression” is the only EQ‐5D dimension showing convergent validity with cognition, responsiveness results diverge from prior expectations, raising questions about convergent validity.^1^

Overall, proxy reports show strengths in “mobility,” “self‐care,” and “usual activities” dimensions, and it is recommended to rely on proxies for these observable dimensions. However, challenges remain in accurately capturing the more subjective dimensions like “pain/discomfort” and “anxiety/depression.” These difficulties have been noted in the literature, where proxies often face challenges in assessing subjective dimensions.^9^, ^23^, ^27^

Results suggest PwD self‐reports are optimal for certain dimensions, while proxy reports are recommended for others, depending on PwD's residential status. For “pain/discomfort” and “anxiety/depression,” PwD reports are most appropriate due to the challenges of proxy reporting on subjective experiences.^27^ In contrast, proxy reports demonstrate stronger responsiveness and validity for observable dimensions, particularly in institutionalized PwD. Staff proxy reports are recommended for institutionalized populations in the “mobility,” “self‐care,” and “usual activities” dimensions. In community‐dwelling populations, there is insufficient evidence to deviate from PwD self‐report for “mobility,” making it the only dimension with differing recommendations based on residential setting. Informal proxies may be more appropriate for community‐dwelling populations. These findings are summarized in Table 5, which presents the recommended respondent for each EQ‐5D dimension based on the PwD's residential status. These recommendations can guide the integration of PwD and proxy EQ‐5D reports into a comprehensive utility score for use in dementia economic evaluations.

**TABLE 5:** EQ‐5D target dimension level respondent by residential status of PwD.

	Residential setting of PwD	
EQ‐5D dimension	*Community dwelling*	*Institutionalized*
Mobility	PwD	Staff proxy
Self‐care	Informal proxy	Staff proxy
Usual activities	Informal proxy	Staff proxy
Pain/discomfort	PwD	PwD
Anxiety/depression	PwD	PwD

Abbreviation: PwD, people with dementia.

### 4.5 Limitations

While our study provides valuable insights, several limitations warrant acknowledgment. The dataset's predominance in care home settings limits generalizing findings to PwD living in the community, which may be particularly relevant for economic evaluations, for which outcomes can vary significantly by care setting. We could not distinguish dementia severity stages due to limited observations from people with severe dementia and increased data missingness, restricting findings to mild‐to‐moderate stages; therefore, different recommendations may be warranted for severe stages. Additionally, the data were not presented in a manner that allowed analysis of non‐completion rates, such as cases in which no survey was returned or the respondent passed away. Completion rates for both PwD and informal proxies were low in the EPIC trial, reflecting the data collection process beyond our control. Uncertainty surrounding the “usual activities” dimension's interpretation necessitates further investigation. Observed ceiling effects in PwD reports and strong proxy responsiveness suggest future research could investigate these discrepancies and strategies to enhance proxy reporting reliability for this dimension. This research involved judgments regarding the optimal respondent and accuracy criteria. Future studies could incorporate qualitative research to explore these aspects further.

## 5 CONCLUSION

This study uses a novel, extensive dataset, making it one of the largest in research on this topic. The dataset spans community‐dwelling and institutionalized populations from dementia trials. While differences in study designs and data collection methods introduce complexities, similarities in intervention types, data collection time points, and instruments collected may mitigate these challenges. This substantial dataset strengthens the reliability of our findings, providing valuable insights into the nuances associated with administration methods and proxy selection in assessing HRQoL within dementia populations via EQ‐5D. Our analysis identifies recommendations to refine data collection and enhance HRQoL assessment validity in dementia. Insights from psychometric analysis, inter‐rater agreement, feasibility, and acceptability have been integrated to propose a composite approach to EQ‐5D dimension reporting. This approach identifies the most effective respondent, whether PwD or proxy, for each dimension, offering a potential method for improving HRQoL assessment accuracy in future economic evaluations. This approach is potentially more reflective of dementia experience than relying solely on either respondent.

## CONFLICT OF INTEREST STATEMENT

All authors declare no conflicts of interest. Author disclosures are available in the supporting information.

## CONSENT STATEMENT

This study used secondary data, and direct consent from participants was not required. However, informed consent was obtained from participants in the original trials, which were conducted in accordance with ethical guidelines and regulatory requirements.

## Supporting information



Supporting Information

Supporting Information

## References

[1] Hussain H , Keetharuth A , Rowen D , Wailoo A . Convergent validity of EQ‐5D with core outcomes in dementia: a systematic review. Health Qual Life Outcomes. 2022;20(1):1‐18. 36403046 10.1186/s12955-022-02062-1 PMC9675120

[2] Harding AJ , Morbey H , Ahmed F , et al. A core outcome set for nonpharmacological community-based interventions for people living with dementia at home: a systematic review of outcome measurement instruments. Gerontologist. 2021;61(8):e435‐e448. 32583858 10.1093/geront/gnaa071 PMC8599310

[3] Webster L , Groskreutz D , Grinbergs‐Saull A , et al. Core outcome measures for interventions to prevent or slow the progress of dementia for people living with mild to moderate dementia: systematic review and consensus recommendations. PLoS One. 2017;12(6):e0179521. 28662127 10.1371/journal.pone.0179521 PMC5491018

[4] Smith S , Lamping D , Banerjee S , et al. Development of a new measure of health‐related quality of life for people with dementia: DEMQOL. Psychol Med. 2007;37(5):737 10.1017/S0033291706009469 [published Online First: 2006/12/21] 17176501

[5] Logsdon RG , Gibbons LE , McCurry SM , Teri L . Quality of life in Alzheimer's disease: patient and caregiver reports. J Mental Health Aging. 1999;5:21‐32.

[6] NICE . Updated guide to the methods of technology appraisal. Secondary updated guide to the methods of technology appraisal. 2013. Accessed February 13. https://www.nice.org.uk/process/pmg9/chapter/foreword

[7] Li L , Nguyen K‐H , Comans T , Scuffham P . Utility‐based instruments for people with dementia: a systematic review and meta‐regression analysis. Value in Health. 2018;21(4):471‐81. 29680105 10.1016/j.jval.2017.09.005

[8] Keetharuth AD , Hussain H , Rowen D , Wailoo A . Assessing the psychometric performance of EQ‐5D‐5L in dementia: a systematic review. Health Qual Life Outcomes. 2022;20(1):139. 36171595 10.1186/s12955-022-02036-3 PMC9520934

[9] Hounsome N , Orrell M , Edwards RT . EQ‐5D as a quality of life measure in people with dementia and their carers: evidence and key issues. Value Health. 2011;14(2):390‐99. 21402307 10.1016/j.jval.2010.08.002

[10] National Institute for Health and Care Excellence (NICE) . Introduction to health technology evaluation. In: NICE Health Technology Evaluations: The Manual. Accessed February 13, 2025. https://www.nice.org.uk/process/pmg36/chapter/introduction-to-health-technology-evaluation

[11] Orgeta V , Edwards RT , Hounsome B , Orrell M , Woods B . The use of the EQ‐5D as a measure of health‐related quality of life in people with dementia and their carers. Qual Life Res. 2015;24(2):315‐24. 25129054 10.1007/s11136-014-0770-0 PMC4317511

[12] Lin X‐J , Lin I‐M , Fan S‐Y . Methodological issues in measuring health‐related quality of life. Tzu Chi Med J. 2013;25(1):8‐12.

[13] Woods RT , Bruce E , Edwards R , et al. REMCARE: reminiscence groups for people with dementia and their family caregivers‐effectiveness and cost‐effectiveness pragmatic multicentre randomised trial. Health Technol Assess. 2012;16(48):v‐xv. 10.3310/hta16480 23211271

[14] Kerpershoek L , De Vugt M , Wolfs C , et al. Access to timely formal dementia care in Europe: protocol of the Actifcare (ACcess to Timely Formal Care) study. BMC Health Serv Res. 2016;16(1):1‐7. 27550084 10.1186/s12913-016-1672-3 PMC4994155

[15] Surr CA , Holloway I , Walwyn RE , et al. Dementia Care Mapping™ to reduce agitation in care home residents with dementia: the EPIC cluster RCT. Health Technol Assess (Winchester, England). 2020;24(16):1. 10.3310/hta24160 PMC7132533 32216870

[16] Morris JC . The clinical dementia rating (CDR): current version and. Young. 1991;41:1588‐92. 10.1212/wnl.43.11.2412-a 8232972

[17] Ankri J , Beaufils B , Novella J‐L , et al. Use of the EQ‐5D among patients suffering from dementia. J Clin Epidemiol. 2003;56(11):1055‐63. 14614996 10.1016/s0895-4356(03)00175-6

[18] Landis JR , Koch GG . The measurement of observer agreement for categorical data. Biometrics. 1977:159‐74. 843571

[19] Lohr KN . Assessing health status and quality‐of‐life instruments: attributes and review criteria. Qual Life Res. 2002;11(3):193‐205. 12074258 10.1023/a:1015291021312

[20] Cohen J . Statistical Power Analysis for the Behavioral Sciences. Academic Press; 2013.

[21] Buchholz M , Engel L , Kleinke F , et al. Discrepancies between self‐and proxy‐rated quality of life in people living with dementia. Alzheimer Dement. 2024;10(2):e12486. 10.1002/trc2.12486 PMC11186300 38899046

[22] Mitnitski AB , Graham JE , Mogilner AJ , Rockwood K . The rate of decline in function in Alzheimer's disease and other dementias. J Gerontol A: Biomed Sci Med Sci. 1999;54(2):M65‐M69. 10.1093/gerona/54.2.m65 10051857

[23] Rand SE , Caiels J . Using proxies to assess quality of life: a review of the issues and challenges. 2015.

[24] Shearer J , Green C , Ritchie CW , Zajicek JP . Health state values for use in the economic evaluation of treatments for Alzheimer's disease. Drugs Aging. 2012;29(1):31‐43. 22191721 10.2165/11597380-000000000-00000

[25] Coucill W , Bryan S , Bentham P , Buckley A , Laight A . EQ‐5D in patients with dementia: an investigation of inter‐rater agreement. Med Care. 2001;39(8):760‐771. 11468496 10.1097/00005650-200108000-00003

[26] Jönsson L , Andreasen N , Kilander L , et al. Patient‐and proxy‐reported utility in Alzheimer disease using the EuroQoL. Alzheimer Dis Assoc Disord. 2006;20(1):49‐55. 16493236 10.1097/01.wad.0000201851.52707.c9

[27] Bonfiglio V , Umegaki H , Kuzuya M . Quality of life in cognitively impaired older adults. Geriatr Gerontol Int. 2019;19(10):999‐1005. 31436029 10.1111/ggi.13759

