# Supplementary material for: Enhancing HRQoL assessment for economic evaluation in dementia populations
Source: Alzheimers Dement (N Y). 2025 Mar 10;11(1):e70061. doi: 10.1002/trc2.70061 (PMC11891564; doi:10.1002/trc2.70061)
Supplement: Supplementary file 1 — Supporting Information [file TRC2-11-e70061-s002.docx]

**Supplementary material**

***EQ-5D***

EQ-5D is a commonly used generic preference-weighted measure of HRQoL. The descriptive system of EQ-5D includes five dimension items measuring: “mobility”, “self-care”, “usual activities”, “pain/discomfort” and “anxiety/depression” {EuroQol Research Foundation, 2018 #128}. There are two versions of EQ-5D: EQ-5D-3L {EuroQol Group, 1990 #5} and EQ-5D-5L {Herdman, 2011 #129}, which contain the same five dimensions but with three or five levels of response options respectively. EQ-5D can be self-completed or administered by an interviewer by asking respondents to choose the severity level in each dimension that best reflects their health status on that day.

The dimension-item responses can be converted to an index score which can then be used in economic evaluations. The value set for England has been used for EQ-5D-3L data {Dolan, 1997 #197}, and where EQ-5D-5L data has been collected, index scores are calculated using the mapping algorithm conditional on age and gender of the PwD currently recommended by NICE {Hernández Alava, 2023 #253}.

Table S1) Descriptive statistics of the three dementia study samples

|  | Study name | | |
| --- | --- | --- | --- |
| n (%) | REMCARE | EPIC | ACTIFCARE |
| Number of dyads | 488 | 734 | 451 |
| Residential status of PwD  Community dwelling  Institutionalised | 488 (100) | 734 (100) | 426 (94.5)  25 (5.5) |
| Sex of PwD  Male  Female | 245 (50.3)  242 (49.7) | 188 (26.2)  529 (73.8) | 205 (45.5)  246 (54.5) |
| Age of PwD  Mean (SD)  Range | 77.5 (7.3)  54 – 95 | 85.6 (7.6)  58 – 102 | 77.8 (7.9)  47 – 98 |
| Sex of informal proxy  Male  Female | 160 (33.0)  325 (67.0) | - | 151 (33.6)  299 (66.4) |
| Age of informal proxy  Mean (SD)  Range | 69.6 (11.6)  23 – 91 | - | 66.4 (13.3)  25 – 92 |
| Relationship of informal proxy  Spouse  Adult child  Other relative  Friend  Other | 345 (71.0)  101 (20.8)  10 (2.1)  15 (3.1)  15 (3.1) | n=171  44 (25.7)  99 (57.9)  21 (12.3)  7 (4.1) | 287 (63.6)  144 (31.9)  14 (3.1)  4 (<1)  1 (<1) |
| CDR stage  0, no dementia  0.5, very mild  1, mild  2, moderate  3, severe | 30 (6.2)  328 (67.4)  129 (26.5) | 3 (<1)  40 (5.5)  180 (25.0)  271 (37.5)  228 (31.6) | 9 (2.0)  345 (78.2)  87 (19.7) |
| Dementia type  Alzheimer’s disease  Vascular dementia  Dementia with Lewy bodies  Mixed  Unknown  Not reported | 106 (21.8)  24 (5.0)  1 (0.2)  17 (3.5)  36 (7.3)  304 (62.2) | - | 218 (48.6)  52 (11.6)  6 (1.3)  56 (12.5)  90 (20.0)  27 (6.0) |
| MMSE  Mean (SD)  Range | - | - | 19.0 (5.0)  3 –30 |
| Function  BADLS: Mean (SD); Range  IADL: Mean (SD); Range  PSMS: Mean (SD); Range  FAST stage: 1 – 7 | 16.1 (9.6); 0-47 | 1-3: 6 <1  4: 97 (13.6);  5: 75 (10.5);  6: 387 (54.4);  7: 147 (20.7) | 5.2 (2.2); 0-8  3.6 (1.9); 0 -6 |
| Behaviour/mood  CSDD: Mean (SD); Range  NPI-NH: Mean (SD); Range  NPI-Q: Mean (SD); Range | 6.96 (5.0); 0-23 | 12.2 (13.0); 0-84 | 17.9 (18.5); 0-124 |
| BADLS, Bristol activities of daily living scale; CDR, clinical dementia rating scale; CSDD, Cornell scale for depression in dementia; FAST, Functional assessment staging tool; IADL, Lawton’s instrument activities of daily living scale; MMSE, mini-mental state examination; NPI-Q, neuropsychiatric inventory questionnaire; NPI-NH, neuropsychiatric inventory nursing home version; PSMS, physical self-maintenance scale; PwD, person with dementia; SD, standard deviation | | | |

Table S2) Informal proxy EQ-5D administration method: EPIC trial data

| Administration method | n (%) |
| --- | --- |
| Face-to-face | 122 (35.2) |
| Telephone | 21 (6.0) |
| Postal | 203 (58.8) |

Table S3) EQ-5D administration method by informal proxy type: EPIC trial data

| n (%) | Relationship of informal proxy to PwD | | | |
| --- | --- | --- | --- | --- |
| Administration method | Spouse | Offspring | Other relative | Friend |
| Face-to-face | 52 (55.3) | 53 (24.8) | 15 (34.9) | 2 (18.1) |
| Telephone | 3 (3.1) | 16 (7.4) | 2 (4.7) | - |
| Postal | 39 (41.4) | 145 (67.8) | 26 (60.4) | 9 (81.9) |
| *Offspring proxy includes adult children and children-in-law | | | | |

Table S4) Visit frequency by proxy type: ACTIFCARE

| n (%) | Spouse/partner | Offspring | Other relative | Other |
| --- | --- | --- | --- | --- |
| Daily | 16 (43.2) | 95 (32.9) | 13 (48.2) | 2 (22.2) |
| Every few days | 17 (46.0) | 119 (41.2) | 3 (11.1) | 3 (33.4) |
| Weekly | 1 (2.7) | 55 (19.0) | 7 (25.9) | 2 (22.2) |
| Fortnightly | 2 (5.4) | 11 (3.8) | 1 (3.7) | 2 (22.2) |
| Monthly | 1 (2.7) | 6 (2.1) | 2 (7.4) |  |
| Other | - | 3 (1.0) | 1 (3.7) |  |
| *Offspring proxy includes adult children and children-in-law | | | | |

To guide decisions regarding which dementia symptom measure to examine against changes in EQ-5D dimension score reports, the convergent validity evidence presented in our previous systematic review has been summarised in Table S5 below.

Table S5) Summary of EQ-5D dimension level convergent validity evidence

| EQ-5D Dimension | Core dementia symptom measure |
| --- | --- |
| Mobility | Function: Basic ADL and mixed  Behaviour/mood: depression and neuropsychiatric disturbance (via NPI) |
| Self-care | Function: Basic ADL and mixed  Behaviour/mood: depression |
| Usual activities | Function: Basic ADL and mixed  Behaviour/mood: depression |
| Pain/ discomfort | Function: Basic ADL |
| Anxiety/ depression | Cognition  Behaviour/mood: depression and neuropsychiatric disturbance (via NPI) |
| ADL, activities of daily living; NPI, neuropsychiatric inventory | |

Table S6) Correlation between EQ-5D dimension reports by CDR stage

| EQ-5D dimension | PwD-informal proxy | | |
| --- | --- | --- | --- |
|  | Mild CDR | Moderate CDR | Severe CDR |
| Mobility | 0.60* | 0.53* | 0.41* |
| Self-care | 0.43* | 0.34* | 0.20 |
| Usual activities | 0.33* | 0.24* | 0.04 |
| Pain/discomfort | 0.43* | 0.39* | 0.38* |
| Anxiety/depression | 0.32* | 0.26* | 0.02 |
|  | PwD-staff proxy | | |
| Mobility | 0.44* | 0.30* | 0.30* |
| Self-care | 0.16* | 0.06 | 0.00 |
| Usual activities | 0.18* | 0.24* | 0.10 |
| Pain/discomfort | 0.29* | 0.21* | -0.05 |
| Anxiety/depression | 0.18* | 0.19* | 0.18* |
| *p<0.05; correlation used is spearman’s rank; PwD, person with dementia; CDR, clinical dementia rating | | | |

Table S7) Analysis of Directional Changes in EQ-5D-5L Dimensions Relative to Dementia Symptom Measures

| Proportions (%) | | **Change in functional status** | | | **Change in behaviour/mood status** | | | **Change in cognitive status** | | |
| --- | --- | --- | --- | --- | --- | --- | --- | --- | --- | --- |
| Change in EQ-5D-5L dimension reports by different respondents | | No change | Worsened | Improved | No change | Worsened | Improved | No change | Worsened | Improved |
| ***Mobility*** | | | | | | | | | | |
| No change | PwD | ***22.9*** | 63.8 | 13.3 | ***9.6*** | 52.0 | 38.4 |  |  |  |
|  | Proxy | ***27.7*** | 58.3 | 14.0 | ***9.2*** | 44.7 | 46.1 |  |  |  |
|  | Staff | ***59.5*** | 33.3 | 7.2 | ***13.6*** | 40.8 | 45.6 |  |  |  |
| Worsened | PwD | 29.4 | ***61.5*** | 9.1 | 11.0 | ***40.7*** | 48.3 |  |  |  |
|  | Proxy | 24.2 | ***70.5*** | 5.3 | 4.4 | ***54.0*** | 41.6 |  |  |  |
|  | Staff | 52.7 | ***43.2*** | 4.1 | 6.6 | ***44.1*** | 49.3 |  |  |  |
| Improved | PwD | 30.3 | 56.6 | ***13.3*** | 10.3 | 52.6 | ***37.1*** |  |  |  |
|  | Proxy | 16.7 | 61.7 | ***21.6*** | 9.5 | 47.6 | ***42.9*** |  |  |  |
|  | Staff | 51.0 | 51.0 | ***11.8*** | 10.7 | 30.4 | ***58.9*** |  |  |  |
| ***Self-care*** | | | | | | | | | | |
| No change | PwD | ***24.4*** | 61.2 | 14.4 | ***10.2*** | 47.2 | 42.7 |  |  |  |
|  | Proxy | ***34.3*** | 50.8 | 14.9 | ***11.6*** | 47.3 | 41.1 |  |  |  |
|  | Staff | ***60.6*** | 31.2 | 8.2 | ***9.6*** | 34.8 | 55.6 |  |  |  |
| Worsened | PwD | 28.7 | ***63.0*** | 8.3 | 9.2 | ***50.5*** | 40.3 |  |  |  |
|  | Proxy | 13.3 | ***79.8*** | 6.9 | 3.9 | ***49.4*** | 46.7 |  |  |  |
|  | Staff | 48.4 | ***49.7*** | 1.9 | 9.6 | ***50.9*** | 39.5 |  |  |  |
| Improved | PwD | 27.3 | 63.6 | ***9.1*** | 11.1 | 60.0 | ***28.9*** |  |  |  |
|  | Proxy | 30.0 | 43.3 | ***26.7*** | 3.9 | 46.7 | ***50.0*** |  |  |  |
|  | Staff | 60.7 | 25.0 | ***14.3*** | 15.6 | 31.3 | ***53.1*** |  |  |  |
| ***Usual activities*** | | | | | | | | | | |
| No change | PwD | ***28.0*** | 57.8 | 41.2 | ***11.7*** | 49.1 | 39.2 |  |  |  |
|  | Proxy | ***29.4*** | 55.0 | 15.6 | ***8.3*** | 44.4 | 47.3 |  |  |  |
|  | Staff | ***52.8*** | 37.6 | 9.6 | ***11.9*** | 39.4 | 48.7 |  |  |  |
| Worsened | PwD | 27.0 | ***63.1*** | 9.9 | 6.8 | ***50.8*** | 42.4 |  |  |  |
|  | Proxy | 18.6 | ***73.5*** | 7.9 | 7.6 | ***52.5*** | 39.9 |  |  |  |
|  | Staff | 57.8 | ***37.0*** | 5.2 | 6.6 | ***46.0*** | 47.4 |  |  |  |
| Improved | PwD | 20.2 | 67.9 | ***11.9*** | 10.7 | 46.4 | ***42.9*** |  |  |  |
|  | Proxy | 33.3 | 50.0 | ***16.7*** | 6.3 | 43.7 | ***50.0*** |  |  |  |
|  | Staff | 59.1 | 39.4 | ***1.5*** | 14.5 | 33.3 | ***51.2*** |  |  |  |
| ***Pain/discomfort*** | | | | | | | | | | |
| No change | PwD | ***26.8*** | 60.0 | 13.2 | ***11.1*** | 45.7 | 43.2 |  |  |  |
|  | Proxy | ***28.6*** | 57.8 | 13.6 | ***9.1*** | 47.4 | 43.5 |  |  |  |
|  | Staff | ***54.2*** | 38.3 | 7.5 | ***11.5*** | 41.0 | 47.5 |  |  |  |
| Worsened | PwD | 22.0 | ***64.8*** | 13.2 | 7.1 | ***58.2*** | 34.7 |  |  |  |
|  | Proxy | 22.9 | ***65.7*** | 11.4 | 3.7 | ***52.8*** | 43.5 |  |  |  |
|  | Staff | 58.0 | ***37.7*** | 4.3 | 4.4 | ***50.7*** | 44.9 |  |  |  |
| Improved | PwD | 27.0 | 62.9 | ***10.1*** | 10.5 | 50.5 | ***39.0*** |  |  |  |
|  | Proxy | 18.7 | 70.3 | ***11.0*** | 9.2 | 45.4 | ***45.4*** |  |  |  |
|  | Staff | 57.3 | 36.6 | ***6.1*** | 11.8 | 31.8 | ***56.4*** |  |  |  |
| **Anxiety/depression** | | | | | | | | | | |
| No change | PwD |  |  |  | 12.8 | 46.2 | 41.0 | ***13.2*** | 56.5 | 30.2 |
|  | Proxy |  |  |  | 11.0 | 46.4 | 42.6 | ***10.2*** | 64.2 | 25.6 |
|  | Staff |  |  |  | 13.0 | 39.7 | 47.3 | ***-*** | - | - |
| Worsened | PwD |  |  |  | 3.7 | 61.0 | 35.3 | 7.4 | ***64.8*** | 27.8 |
|  | Proxy |  |  |  | 5.6 | 58.3 | 36.1 | 12.3 | ***50.8*** | 23.9 |
|  | Staff |  |  |  | 2.1 | 70.2 | 27.6 | - | ***-*** | - |
| Improved | PwD |  |  |  | 8.9 | 47.8 | 43.3 | 12.7 | 60.3 | ***27.0*** |
|  | Proxy |  |  |  | 3.1 | 39.2 | 57.7 | 15.9 | 58.0 | ***26.1*** |
|  | Staff |  |  |  | 6.7 | 28.1 | 65.2 | - | - | ***-*** |

Table S8a) Correlation between change in EQ-5D index score and change in dementia symptom measure scores

| Correlation coefficient; CI | | CDR severity stage at T2** | | |
| --- | --- | --- | --- | --- |
| Instrument and rater-type | N | Sum | Mild | Moderate |
| *CDR change*  PwD  Informal proxy  Staff proxy | 420  388  398 | **-0.05**  [-0.14; 0.05]  -**0.21***  [-0.30; -0.11]  **-0.26***  [-0.35; -0.17] | 0.01  0.05  -0.22 | 0.03  -0.08  -0.07 |
| *MMSE change*  PwD  Informal proxy | 263  269 | -**0.02**  [-0.14; 0.10]  **0.07**  [-0.05; 0.19] | 0.10  0.06 | -0.16  0.03 |
| *NPI-NH change*  PwD  Informal proxy  Staff proxy | 126  58  398 | **-0.02**  [-0.19; 0.16]  **-0.04**  [-0.29; 0.23]  **-0.16***  [-0.25; -0.06] | 0.06  -  -0.11 | -0.13  -  -0.26* |
| *NPI-Q change*  PwD  Informal proxy | 296  345 | **-0.03**  [-0.14; 0.08]  **-0.23***  [-0.33; -0.13] | 0.09  -0.20* | -0.09  -0.39* |
| *CSDD change*  PwD  Informal proxy | 212  229 | **-0.01**  [-0.14; 0.13]  **-0.17***  [-0.30; -0.05] | -0.06  -0.25* | 0.08  -0.03 |
| *BADLS change*  PwD  Informal proxy | 276  307 | **-0.18***  [0.06; 0.29]  **-0.42***  [0.32; 0.51] | -0.12  -0.42* | -0.34*  -0.38* |
| *IADL-PSMS change*  PwD  Informal proxy | 282  328 | **-0.03**  [-0.15; 0.08]  **0.33**  [0.23; 0.42] | -0.02  0.31* | -0.04  0.17 |
| *FAST change*  PwD  Informal proxy  Staff proxy | 121  56  378 | **0.07**  [-0.11; 0.24]-**0.14***  [-0.39; 0.13]  **-0.12***  [-0.21; -0.02] | 0.10  0.08  -0.23 | 0.05  -0.23  -0.08 |
| **CDR at T0 for CSDD and BADLS assessments due to lack of follow-up CDR data in REMCARE | | | | |

Table S8b) Correlation between change in mobility dimension score and change in dementia symptom measure scores

| Correlation coefficient; CI | | CDR severity stage at T2** | | |
| --- | --- | --- | --- | --- |
| Instrument and rater-type | N | Sum | Mild | Moderate |
| *CDR change*  PwD  Informal proxy  Staff proxy | 439  397  396 | **0.07**  [-0.02; 0.16]  **0.11***  [0.02; 0.21]  **0.20***  [0.10; 0.29] | -0.10  -0.01  0.19 | 0.07  0.01  -0.00 |
| *BADLS change*  PwD  Informal proxy | 289  311 | **0.16***  [-0.27; -0.04]  **0.24***  [-0.34; -0.13] | 0.11  0.22* | 0.40*  0.25* |
| *IADL-PSMS change*  PwD  Informal proxy | 294  334 | **0.01**  [-0.11; 0.12]  **-0.23***  [-0.33; -0.13] | 0.03    -0.22* | 0.02    -0.10 |
| *FAST change*  PwD  Informal proxy  Staff proxy | 129  61  379 | **-0.13**  [-0.30; 0.05]  **0.07**  [-0.18; 0.32]  **0.09**  [-0.01; 0.19] | -0.13  -0.25  0.30* | -0.09  0.10  -0.06 |
| *NPI-NH change*  PwD  Informal proxy  Staff proxy | 134  63  399 | **-0.12**  [-0.28; 0.05]  **-0.06**  [-0.19; 0.30]  **-0.02**  [-0.12; -0.08] | -0.12  -  -0.01 | -0.06  -  -0.01 |
| *NPI-Q change*  PwD  Informal proxy | 310  351 | **-0.01**  [-0.12; 0.10]  **-0.03**  [-0.13; 0.08] | -0.14*  0.03 | 0.07  0.07 |
| *CSDD change*  PwD  Informal proxy | 221  238 | **0.04**  [-0.09; 0.17]  **0.14***  [0.01; 0.27] | 0.12  0.16* | -0.14  0.09 |
| **CDR at T0 for CSDD and BADLS assessments due to lack of follow-up CDR data in REMCARE | | | | |

Table S8c) Correlation between change in self-care dimension score and change in dementia symptom measure scores

| Correlation coefficient; CI | | CDR severity stage at T2** | | |
| --- | --- | --- | --- | --- |
| Instrument and rater-type | N | Sum | Mild | Moderate |
| *CDR change*  PwD  Informal proxy  Staff proxy | 435  397  396 | **0.07**  [-0.03; 0.16]  **0.29***  [0.20; 0.38]  **0.32***  [0.23; 0.41] | -0.01  0.11  0.28* | -0.01  0.05  0.15 |
| *BADLS change*  PwD  Informal proxy | 288  311 | **0.14***  [-0.25; -0.03]  **0.47***  [-0.55; -0.38] | 0.12  0.50* | 0.21  0.39* |
| *IADL-PSMS change*  PwD  Informal proxy | 293  334 | **-0.02***  [-0.13; -0.10]  **-0.46***  [-0.55; -0.38] | -0.08    -0.39* | -0.06    -0.34* |
| *FAST change*  PwD  Informal proxy  Staff proxy | 127  61  379 | **-0.03**  [-0.20; 0.15]  **0.26***  [0.01; 0.48]  **0.26***  [0.16; 0.35] | 0.04  -  0.37* | -0.09  -  0.19* |
| **CDR at T0 for BADLS assessments due to lack of follow-up CDR data in REMCARE | | | | |

Table S8d) Correlation between change in usual activities dimension score and change in dementia symptom measure scores

| Correlation coefficient; CI | | CDR severity stage at T2** | | |
| --- | --- | --- | --- | --- |
| Instrument and rater-type | N | Sum | Mild | Moderate |
| *CDR change*  PwD  Informal proxy  Staff proxy | 427  397  396 | **0.02**  [-0.08; 0.11]  **0.18***  [0.09; 0.28]  **0.19***  [0.10; 0.29] | -0.15*  0.11  0.06 | 0.05  0.08  0.18* |
| *BADLS change*  PwD  Informal proxy | 283  308 | **0.19***  [-0.30; -0.08]  **0.30***  [-0.40; -0.20] | 0.17*  0.33* | 0.27*  0.23* |
| *IADL-PSMS change*  PwD  Informal proxy | 286  333 | **-0.01**  [-0.12; 0.11]  **-0.31***  [-0.41; -0.21] | 0.05    -0.19* | -0.04    -0.27* |
| *FAST change*  PwD  Informal proxy  Staff proxy | 125  61  379 | **-0.08**  [-0.25; 0.10]  **0.08**  [-0.18; 0.32]  **0.03**  [-0.07; 0.13] | -0.17  -  0.01 | 0.00  -  0.14 |
| *CSDD change*  PwD  Informal proxy | 217  231 | **0.03**  [-0.10; 0.16]  **0.10**  [-0.03; 0.22] | -0.01  0.12 | 0.13  0.05 |
| **CDR at T0 for CSDD and BADLS assessments due to lack of follow-up CDR data in REMCARE | | | | |

Table S8e) Correlation between change in pain/discomfort dimension score and change in dementia symptom measure scores

| Correlation coefficient; CI | | CDR severity stage at T2** | | |
| --- | --- | --- | --- | --- |
| Instrument and rater-type | N | Sum | Mild | Moderate |
| *CDR change*  PwD  Informal proxy  Staff proxy | 431  394  395 | **0.00**  [-0.09; 0.10]  **-0.03**  [-0.13; 0.07]  **-0.04**  [-0.14; 0.06] | -0.01  -0.04  0.01 | -0.03  0.05  -0.12 |
| *BADLS change*  PwD  Informal proxy | 289  310 | **0.08***  [-0.19; 0.04]  **0.09***  [-0.20; 0.02] | 0.05  0.05 | 0.17  0.18 |
| *IADL-PSMS change*  PwD  Informal proxy | 287  332 | **0.08**  [-0.04; 0.19]  **-0.02***  [-0.13; 0.09] | 0.02    -0.10 | 0.11    -0.05 |
| *FAST change*  PwD  Informal proxy  Staff proxy | 126  60  378 | **0.09**  [-0.09; 0.26]  **-0.08**  [-0.33; 0.17]  **-0.03**  [-0.13; 0.08] | 0.07  -  -0.02 | 0.05  -  -0.06 |
| *CSDD change*  PwD  Informal proxy | 220  233 | **-0.03**  [-0.17; 0.10]  **0.08**  [-0.05; 0.21] | -0.03  0.10 | -0.05  0.04 |
| **CDR at T0 for CSDD and BADLS assessments due to lack of follow-up CDR data in REMCARE | | | | |

Table S8f) Correlation between change in anxiety/depression dimension score and change in dementia symptom measure scores

| Correlation coefficient; CI | | CDR severity stage at T2** | | |
| --- | --- | --- | --- | --- |
| Instrument and rater-type | N | Sum | Mild | Moderate |
| *CDR change*  PwD  Informal proxy  Staff proxy | 433  395  395 | **-0.03**  [-0.12; 0.07]  **0.02**  [-0.08; 0.12]  **0.06**  [-0.04; 0.15] | -0.04  -0.18*  0.26* | -0.02  0.13  0.15 |
| *MMSE change*  PwD  Informal proxy | 269  271 | **-0.20***  [-0.14; 0.10]  **0.08***  [-0.04; 0.20] | -0.07  0.02 | 0.21  0.39* |
| *NPI-NH change*  PwD  Informal proxy  Staff proxy | 132  63  398 | **0.15**  [-0.02; 0.31]  **0.07**  [-0.18; 0.31]  **0.35***  [0.26; 0.44] | 0.01  -  0.28* | 0.27*  -  0.52* |
| *NPI-Q change*  PwD  Informal proxy | 304  348 | **-0.02**  [-0.13; 0.09]  **0.28***  [0.18; 0.37] | -0.10  0.21* | 0.08  0.46* |
| *CSDD change*  PwD  Informal proxy | 218  233 | **0.03**  [-0.10; 0.16]  **0.21***  [0.08; 0.33] | 0.13  0.27* | -0.19  0.09 |
| **CDR at T0 for CSDD assessments due to lack of follow-up CDR data in REMCARE | | | | |

***Factors influencing EQ-5D index scores***

Multivariate linear regression analysis output is presented in Table S9a and S9b and indicates that proxy models generally have higher R-squared values, suggesting a larger proportion of variance in EQ-5D index scores is explained by independent variables in proxy models. Regression coefficients are generally larger in proxy models, with more variables significantly affecting proxy scores compared to PwD-reports. Functional measures consistently predict EQ-5D index scores across all studies, with demographic factors such as proxy sex playing a role, where male proxies are associated with lower proxy EQ-5D scores in REMCARE, and the reverse is true for ACTIFCARE. CDR also significantly predicts proxy EQ-5D index scores in ACTIFCARE and EPIC. However, differences emerge across datasets, with spousal proxies showing unique predictors compared to offspring proxies. Behaviour/mood measures (CSDD and NPI) are significant predictors of proxy index scores, but not for PwD-reports. Additionally, proxies' own HRQoL via EQ-5D is a significant predictor in the REMCARE dataset.

Table S9a) Multivariate linear regression results

| *REMCARE data (EQ-5D-3L)* | | | | |
| --- | --- | --- | --- | --- |
| Measure | Coefficient | Std. err. | *t* | *R^2^* |
| PwD | | | | |
| BADLS | -0.003* | 0.009 | -3.64 | 0.07 |
| CDR | 0.100 | 0.019 | 0.52 |  |
| CSDD | -0.010 | 0.002 | -6.26 |  |
| Carer’s own EQ-5D utility | -0.014 | 0.034 | -0.41 |  |
| Age (PwD) | 0.001 | 0.001 | 0.81 |  |
| Sex (PwD) | -0.006* | 0.016 | -0.36 |  |
| Proxy | | | | |
| BADLS | -0.013* | 0.001 | -18.51 | 0.45 |
| CDR | 0.020 | 0.016 | 1.21 |  |
| CSDD | -0.016* | 0.001 | -11.74 |  |
| Carer’s own EQ-5D utility | 0.101* | 0.030 | 3.41 |  |
| Age (RP) | 0.001* | 0.001 | 2.37 |  |
| Sex (RP) | -0.042* | 0.566 | 13.43 |  |
| *ACTIFCARE data (EQ-5D-5L)* | | | | |
| PwD | | | | |
| IADL | 0.007 | 0.004 | 1.65 | 0.11 |
| CDR | 0.008 | 0.017 | 0.48 |  |
| PSMS | 0.030* | 0.005 | 6.61 |  |
| NPI | 0.000 | 0.000 | 0.45 |  |
| MMSE | -0.005* | 0.001 | -3.44 |  |
| Age (PwD) | -0.003* | 0.001 | -2.98 |  |
| Sex (PwD) | -0.020 | 0.014 | -1.39 |  |
| Proxy | | | | |
| IADL | 0.024* | 0.004 | 6.00 | 0.36 |
| CDR | -0.039* | 0.016 | -2.44 |  |
| PSMS | 0.010* | 0.004 | 9.47 |  |
| NPI | -0.003* | 0.000 | -7.45 |  |
| MMSE | -0.005* | 0.001 | -4.09 |  |
| Age (RP) | 0.001 | 0.000 | 1.05 |  |
| Sex (RP) | 0.051* | 0.013 | 3.82 |  |
| *EPIC data (EQ-5D-5L)* | | | | |
| PwD | | | | |
| CDR | 0.019 | 0.013 | 1.50 | 0.02 |
| NPI | -0.001 | 0.001 | -1.88 |  |
| FAST | -0.009 | 0.011 | -0.87 |  |
| Age (PwD) | 0.003* | 0.001 | 2.43 |  |
| Sex (PwD) | -0.021 | 0.020 | -1.08 |  |
| Informal proxy | | | | |
| CDR | -0.100* | 0.028 | -3.47 | 0.22 |
| NPI | -0.000 | 0.002 | -0.29 |  |
| FAST | -0.114* | 0.024 | -4.83 |  |
| Age (PwD) | -0.002 | 0.002 | -0.78 |  |
| Sex (PwD) | -0.081* | 0.041 | -1.96 |  |
| Staff proxy | | | | |
| CDR | -0.135* | 0.014 | -9.79 | 0.22 |
| NPI | -0.000 | 0.001 | -0.04 |  |
| FAST | -0.131* | 0.013 | -10.21 |  |
| Age (PwD) | -0.001 | 0.001 | -0.59 |  |
| Sex (PwD) | -0.024 | 0.021 | -1.16 |  |
| *p<0.05; BADLS, Bristol activities of daily living scale; CDR, clinical dementia rating scale; CSDD, Cornell scale for depression in dementia; FAST, functional assessment staging tool for Dementia; IADL, Lawton instrumental activities of daily living scale; MMSE, mini-mental state examination; NPI, neuropsychiatric inventory; PSMS, physical self-maintenance scale; RP, informal/relative proxy | | | | |

Table S9b) Predictors of EQ-5D index score by informal proxy type

|  | *Coefficient* | Std. err. | *t* | *R^2^* | *Coefficient* | Std. err. | *t* | *R^2^* |
| --- | --- | --- | --- | --- | --- | --- | --- | --- |
| REMCARE | | | | | | | | |
| Spousal proxy | | | | 0.50 | **Offspring proxy** | | | 0.36 |
| BADLS | -0.014* | 0.001 | -17.06 |  | -0.011* | 0.002 | -6.04 |  |
| CDR | 0.023 | 0.029 | 1.22 |  | 0.033 | 0.037 | 0.91 |  |
| CSDD | -0.018* | 0.003 | -10.58 |  | -0.015* | 0.003 | -5.54 |  |
| Carer’s own EQ-5D utility | 0.096* | 0.051 | 2.86 |  | 0.221* | 0.073 | 3.03 |  |
| Age (carer) | -0.003* | 0.002 | -2.49 |  | 0.002 | 0.002 | 0.86 |  |
| Sex (carer) | -0.012* | 0.026 | -2.35 |  | 0.038 | 0.037 | 1.03 |  |
| ACTIFCARE | | | | | | | | |
| IADL | 0.028* | 0.005 | 5.30 | 0.42 | 0.024* | 0.007 | 3.65 | 0.32 |
| CDR | -0.040* | 0.019 | -2.10 |  | -0.002 | 0.029 | 0.04 |  |
| PSMS | 0.048* | 0.005 | 8.63 |  | 0.026* | 0.007 | 3.65 |  |
| NPI | -0.003* | 0.000 | -6.62 |  | -0.003* | 0.008 | -4.43 |  |
| MMSE | -0.006* | 0.002 | -3.90 |  | -0.003 | 0.003 | -1.33 |  |
| Age (carer) | -0.001 | 0.001 | 3.42 |  | -0.003* | 0.001 | -2.30 |  |
| Sex (carer) | 0.057* | 0.017 | -0.90 |  | 0.049* | 0.025 | 2.01 |  |
| EPIC | | | | | | | | |
| CDR | -0.126* | 0.063 | -2.01 | 0.31 | -0.126* | 0.033 | -3.77 | 0.24 |
| NPI-NH | -0.003 | 0.003 | -0.95 |  | -0.001 | 0.002 | -0.60 |  |
| FAST | -0.174* | 0.060 | -2.93 |  | -0.087* | 0.027 | -3.23 |  |
| *p<0.05; BADLS, Bristol activities of daily living scale; CDR, clinical dementia rating scale; CSDD, Cornell scale for depression in dementia; IADL, Lawton’s instrumental activities of daily living scale; FAST, functional assessment staging tool for dementia; MMSE, mini-mental state examination; NPI, neuropsychiatric inventory; PSMS, physical self-maintenance scale; EQ-5D-3L for REMCARE; EQ-5D-5L for ACTIFCARE and EPIC | | | | | | | | |
